# Anatomy meets dentistry! *Linking anatomy and clinical practice in the preclinical dental curriculum*

**DOI:** 10.1186/s12909-016-0825-8

**Published:** 2016-11-25

**Authors:** Nicole Rafai, Martin Lemos, Lieven Nils Kennes, Ayichah Hawari, Susanne Gerhardt-Szép, Irmgard Classen-Linke

**Affiliations:** 1 Department of Prosthodontics and Biomaterials, Center for Implantology, Medical Faculty, RWTH Aachen University, Pauwelsstrasse 30, Aachen, 52074 Germany; 2 Audiovisual Media Center, Medical Faculty, RWTH Aachen University, Pauwelsstrasse 30, Aachen, 52074 Germany; 3 Department of Economics and Business Administration, University of Applied Sciences Stralsund, Zur Schwedenschanze 15, Stralsund, 18435 Germany; 4 Institute of Molecular and Cellular Anatomy, Medical Faculty, RWTH Aachen University, Wendlingweg 2, Aachen, 52074 Germany; 5 Department of Operative Dentistry, Center for Dentistry and Oral Medicine (Carolinum), Medical Faculty, Goethe University of Frankfurt am Main, Theodor-Stern-Kai 7, Frankfurt am Main, 60596 Germany; 6 Present address: Department of Oral and Maxillofacial Surgery, Medical Faculty, RWTH Aachen University, Pauwelsstrasse 30, Aachen, 52074 Germany

**Keywords:** Interdisciplinarity, Anatomy, Dentistry, Clinical skills, Peer-teaching, E-learning, OSCE, Randomized controlled trial, Learning outcomes

## Abstract

**Background:**

Establishing a strong link early on between preclinical coursework and the clinical context is necessary for students to be able to recognize the practical relevance of the curriculum during their preclinical anatomical courses and to transfer knowledge more easily. Our objective was to enhance the clinical relevance of a preclinical anatomy course for second-year medical students of dentistry by implementing an interdisciplinary skills training course on “Palpation of the Head and Neck Muscles” and to measure the learning outcomes.

**Methods:**

For the curricular development of the expanded course module, Kern’s 6-step approach was applied including subjective evaluation. We used a peer-teaching format supported by an e-learning application. A randomized control study measured effects of the two components (skills training, e-module) on learning outcomes. Four learning methods were compared: (1) lecture, (2) lecture + e-module, (3) lecture + skills training, (4) lecture + skills training + e-module. An objective structured clinical examination (OSCE) was used to measure and compare learning outcomes.

**Results:**

The two-way variance analysis demonstrated that participation in the skills training had a statistically significant effect on the OSCE results (*p* = 0.0007). Students who participated in the skills training did better (φ 107.4 ± 14.4 points) than students who only attended the lecture (φ 88.8 ± 26.2 points). Students who used the e-module but did not attend the skills training earned a slightly but not significantly higher average number of points (φ 91.8 ± 31.3 points) than those who only attended the lecture. The learning outcomes of the skills training were again significantly increased when the training was combined with the e-module (φ 121.8 ± 21.8 points), thus making it the ideal method for achieving the learning objectives defined in this study.

**Conclusions:**

The “Palpation of the Head and Neck Muscles” interdisciplinary skills training course linking basic anatomical knowledge and clinical skills led to clearly improved learning outcomes for both, anatomical knowledge and clinical skills. The additional use of an e-learning tool (e-module) improved the learning effect.

## Background

Knowledge of anatomy is highly relevant to dentistry; this is especially obvious when performing anesthesiological or surgical procedures and when using various examination methods. For this reason, a solid knowledge of anatomy is fundamental to the dentist’s daily work [1]. Head and neck anatomy is particularly important.

And yet, anatomy courses are in Germany traditionally taught only during the preclinical part of dentistry studies and are frequently detached from the study of other disciplines as well. Anatomy teaching in Germany and other countries is often not integrated into the clinical context at all or it is integrated to only a limited extent [2]. Students therefore often have a hard time determining any practical relevance. Then later, when they begin treating patients in the clinical part of their studies, they must apply the anatomical knowledge they acquired during the preclinical courses. At that point, however, they often have difficulty calling up this basic knowledge [3].

These traditional teaching methods lead to superficial and one-dimensional learning [4–6]. But according to Drake and Pawlina [7], the role of anatomy in medical teaching is changing. The trend is moving away from anatomy as a subject in which facts are taught in isolation and toward an “integrated” anatomy course that aims to prepare the students for their clinical work early in their studies [7].

Establishing a strong link early on between preclinical coursework and the clinical context enables students to recognize the practical relevance of the curriculum during their preclinical courses and to transfer knowledge more easily [8, 9].

The German Medical Licensure Act [10] and the draft of the German Dental Licensure Act from June 2007 emphasize that medical education should “illustrate the links between medical foundations and clinical applications”. At the same time, “the teaching of scientific and theoretical foundations […] must focus on training content relevant to medicine and dentistry”. In addition, the two acts state that teaching should “promote interdisciplinary thinking”. They also foresee “integrated courses in which suitable clinical subjects are included” and linking “the teaching of theoretical and clinical knowledge […] as closely as possible during the entire training period.” [10].

When adapting a curriculum to reflect these requirements and when selecting a suitable teaching method, educators must bear in mind that today’s students are a new generation with new requirements for teaching [11]. Improvements in technology and increasing availability mean that students expect e-learning to be used for teaching [12].

Therefore, in order to teach anatomy in a way that meets all these requirements and to prepare the students for their clinical work early in their studies, we implemented an interdisciplinary skills training seminar and an additional e-learning module during curricular development. Considering relevant findings in educational and scientific aspects of medical teaching we used Kern’s 6-step approach [13], peer teaching [14] and Peyton’s 4-step approach to teaching skills [15].

By using an objective structured clinical examination we measured and compared the learning outcomes and evaluated the results by a two-way analysis of variance.

Main research questions in this context have been:

- The effect of the new course components on the students’ learning outcomes.
- The potential added value of e-learning.
- The correlation between theoretical knowledge and practical skills.
- The assessment of the students’ perception of the clinical relevance of the learning content.

## Methods

We expanded the preclinical head-and-neck anatomy lab for second-year preclinical students to include aspects of clinical teaching. This interdisciplinary approach (dental clinic plus anatomy) was used to illustrate the clinical relevance of anatomical knowledge.

The project was conducted in two stages:

1. Curricular development of the expanded course module using Kern’s 6-step approach, including subjective evaluation [13].
2. Monocentric randomized case–control study in a 4-group design to study the effect of the course modules on learning outcomes.

### Curriculum development

We used Kern’s 6-step approach to develop a curriculum (see Table 1) [13].

**Table 1:** Kern’s 6-step approach

Step	
1	Problem identification and general needs assessment
2	Needs assessment of targeted learners
3	Goals and objectives
4	Educational strategies
5	Implementation
6	Evaluation and feedback

Our primary focus was correcting the lack of clinical relevance to the students of the learning content of the head-and-neck anatomy lab. The defined target group was dentistry students in the second year that had completed the first part of the preclinical anatomy lab that teaches the anatomy of the head and neck. We considered suitable clinical contexts in which (a) second year students without any previous knowledge of dentistry could be taught in a practically relevant manner and (b) the clinical relevance of the knowledge of basic anatomy would be clearly illustrated. Based on these considerations, the lab topic selected was the palpation of muscles. For this topic, knowledge of anatomical principles is directly related to mastery of palpation technique.

#### Formulating learning objectives

For the new course module (linking muscle anatomy with the techniques used to palpate selected muscles), we first developed additional learning objectives for seven clinically relevant head and neck muscles.

Existing learning objectives with respect to anatomy:

The students are able to name and identify each muscle (see Table 2) with its insertion and origin, including innervation, blood supply and function (cognitive competence).

**Table 2:** Selected muscles and associated palpation techniques

Muscle/muscles	Palpation technique
Masseter	extraoral/intraoral
Medial pterygoid	extraoral/intraoral
Lateral pterygoid	intraoral
Temporalis	extraoral/intraoral
Suprahyoid	extraoral/intraoral
Infrahyoid	extraoral
Sternocleidomastoid	extraoral

New learning objectives with respect to the clinical aspect:

The students are able to perform an extraoral and intraoral palpation of each muscle (see Table 2) and acquire the theoretical and practical skills for clinical examination and assessment of the muscles (psychomotoric skills and expertise).

#### Teaching method: blended-learning peer teaching

Based on the learning objectives, we designed a lecture and a skills training seminar entitled “Palpation of the Head and Neck Muscles” to supplement the course that had been offered to date. We decided to use a peer-teaching format for the training. This format offers the advantage of small group sizes (a teacher–student ratio of 1:3 to 1:6) and capitalizes on the ability of peer tutors to communicate more effectively and, in so doing, improve the learning environment [14]. We selected the incremental Peyton method [15] for teaching the practical skills in the training (see Table 3).

**Table 3:** Peyton’s 4-step approach to teaching skills

Step	
1	Demonstration (trainer demonstrates at normal speed, without commentary)
2	Deconstruction (trainer demonstrates while describing steps)
3	Learner comprehension (trainer demonstrates while learner describes steps)
4	Learner performance (learner demonstrates while learner describes steps)

**Fig. 1:**
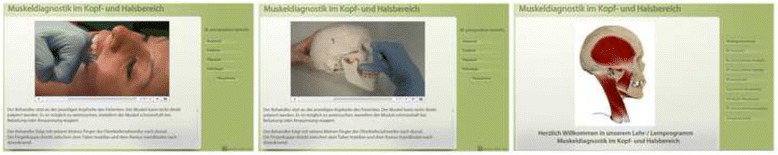
Screenshots of the e-module “Palpation of the Head and Neck Muscles”

To prepare for the skills training, the peer teachers underwent a 2-h peer-training course at the beginning of the week in which the student skills training seminars were scheduled. To prepare for the training, the peer teachers were able to consult the e-module (see below) in which all of the learning content was also presented via videos (see Fig. 1).

During the training, the peer teachers learned to use the Peyton method and worked with the e-module in the training room, with an e-learning monitor located at each dental chair. They practiced the palpation techniques on each other in groups of three under the supervision of an instructor.

#### Teaching material: e-module

An online learning program was developed by the authors in collaboration with the RWTH Aachen University Faculty of Medicine’s Audiovisual Media Center (AVMZ) as supplementary teaching material. The e-module was intended to be used by the students to prepare for and follow up on the skills training. It would also serve to standardize the teaching and learning content. The planned intensive link between the e-module, the lecture, and the skills training led to a true blended-learning concept using a combination of face-to-face teaching and e-learning applications.

The e-module entitled “Palpation of the Head and Neck Muscles” covers all aspects of the selected muscles, including anatomy, pathology, and diagnostics. Texts, graphics, and anatomical illustrations are supplemented by videos demonstrating the individual palpation techniques. The e-module has an explorative structure, meaning users can move about the program freely and are not required to follow any particular order. Basically, the e-module functions like a set of index cards and serves as a kind of reference work. The web-based, flash-based e-module is part of the eMedia Skills Lab, the Faculty of Medicine’s teaching and learning platform. Students at all levels can log into the e-module at any time.

Readers can access the e-module via the following link:

https://emedia-medizin.rwth-aachen.de

Zahnmedizin _ Prothetik_ Muskeldiagnostik im Kopf-und Halsbereich

#### Implementation

The interdisciplinary course including the lecture and the skills training was conducted for the first time in 2010. Second year preclinical students used specimens to learn about the individual muscles and their anatomy during the traditional cadaver dissection course. The new palpation course was offered as an elective course in the middle of the second week of the 5-week head-and-neck anatomy course. As part of the course, the students attended a 45-min lecture on the clinical relevance of the individual muscles, and the individual palpation techniques were presented. During the lecture the students were informed about the e-module and its application.

The students subsequently learned how to perform the individual palpation techniques in a 2-h peer-teaching skills training and practiced on each other. In order to underscore the clinical relevance, the training was held in the same rooms as the students’ clinical treatment courses. The e-module, available at a video workstation, was also used during the seminar to support the teaching.

### Randomized control study

In the second stage, a monocentric randomized case–control study involving four study groups was used to study the new course concept.

The sole study center was the Institute of Molecular and Cellular Anatomy in collaboration with the Department of Prosthodontics and Biomaterials, Center for Implantology of the Medical Faculty, RWTH Aachen University. The study was conducted during the head-and-neck dissection course held in Aachen in 2011. All second-year students who wished to participate in the palpation course were also allowed to participate in the study (inclusion criteria). The course was offered as an elective and the learning outcomes test did not influence the students’ grades for the head-and-neck anatomy course.

The students were randomly assigned to four study groups (see Table 4).

**Table 4:**
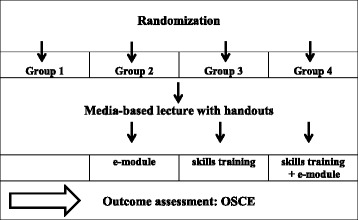
Four study groups

Randomization was performed with the support of the Department of Medical Statistics RWTH Aachen University. There was no relevant difference between the groups according to age, sex, previous knowledge and previous skills training. All of the students participating in the study attended the same 45-min lecture at the beginning of the study which was given by the same experienced lecturer. After the lecture, they were given a handout containing key information about the covered anatomy and palpation techniques. Then, depending on their study group, they were assigned one of four options for enhancing the lecture content and for preparing to take a test on learning outcomes.

The 2-h skills training seminar for groups 3 and 4 took place directly after the lecture. The e-module for groups 2 and 4 was also activated right after the lecture and was available for two days until the examination. Groups 1 and 3 had no access to the e-module, but had the same time to study the anatomy and palpation technique to prepare for the examination by using the detailed handout.

The learning outcomes of each study group were tested by means of an objective structured clinical examination (OSCE) with seven stations. The OSCE was conducted 2 days after the lecture. At each of the seven stations, one muscle was tested in a standardized manner within 2 min. The test consisted of two parts:

1. Demonstrating the palpation technique on simulation patients
2. Describing the anatomical details (insertion, origin, etc.)

The examiners were upper-level students (peer examiners, fourth-year students) who had been trained in advance in testing the content of their circuit station and in using the respective checklist. The examiners did not change stations during the OSCE and were the same for all students. The station checklists were all set up using the same methodology (Part 1 Palpation, Part 2 Anatomy). Prior to the OSCE, the modified Angoff method was used to define the borderlines [16]. The point scores for individual responses were defined in the checklists accordingly. Depending on the complexity of the muscle anatomy and the difficulty of the palpation technique, the maximum number of attainable points per station ranged from 17 to 29 points.

After completing the OSCE, the students were asked to grade how helpful the test-preparation method was on a scale of 1 (*very good*) to 5 (*unsatisfactory*) and to rate the clinical relevance of the learning material (“Was the method relevant?”) according to a 5-level Likert scale from 1 (*definitely*) to 5 (*not at all*). They were also able to add comments.

#### Statistical evaluation

Randomization was performed in blocks of various lengths. Blinding was not possible due to the nature of the study. However, because group assignments were made using student enrollment numbers and the random assignments were binding, distortion and selection bias is ruled out for this study.

A two-way analysis of variance (ANOVA) was used to study the impact of the skills training and the e-module and of the interaction of these components on the OSCE results. Both, the homoscedastic distribution and the normal distribution of residuals were verified in a residual analysis. All statistical analyses were performed with the SAS 9.1 (© 2002–2003 by SAS Institute, Cary, NC, USA) and R (R 2.11.1 © 2010 The R Foundation for Statistical Computing) statistical programs.

## Results

### Effect of the skills training and added value of the e-module: OSCE results

All of the second-year students participated in the study (*N* = 53). The results of one student in group 2 could not be evaluated because the student was unable to access the e-module. Therefore, the results of 52 students were evaluated.

**Fig. 2:**
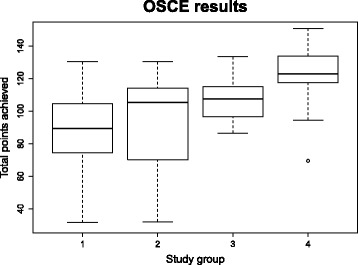
Box plots displaying the distribution of points achieved by OSCE for each group: impact of the influencing factors of skills training (group 3), e-module (group 2) and both (group 4) on the OSCE results (*N* = 52)

The two-way ANOVA demonstrated that participation in the skills training had a statistically significant effect on the OSCE results (*p* = 0.0007). However, no significant effect of the e-module was demonstrated (*p* = 0.2005). Students who participated in the skills training achieved higher learning outcomes (OSCE points achieved: φ 107.4 ± 14.4) than students who only attended the lecture (φ 88.8 ± 26.2 points) or students who attended the lecture and used the e-module but did not attend the skills training (φ 91.8 ± 31.3 points). However, the learning outcomes of the skills training were again significantly increased when the training was combined with the e-module (φ 121.8 ± 21.8 points), making it the ideal method for meeting the learning objectives defined in the head-and-neck anatomy course (see Table 5 and Fig. 2).

**Table 5:** Mean values and standard deviations of the points achieved (OSCE) for each group

		OSCE results	
		mean	SD
GROUP	lecture (1)	88.7500	26.2105
	+ e-module (2)	91.7500	31.2995
	+ skills training (3)	107.4231	14.3945
	+ both (4)	121.8077	21.8084
E-MODULE	no	97.7407	23.0114
	yes	107.3800	31.3597
SKILLS TRAINING	no	90.1346	28.1178
	yes	114.6154	19.5332

### Correlation between theoretical knowledge and practical skills

**Fig. 3:**
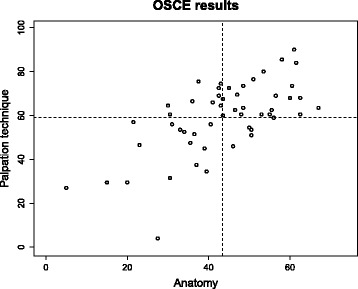
Correlation analysis between anatomical knowledge and palpation skills based on the OSCE results

In order to determine the association between anatomical knowledge and practical skills, a correlation analysis was performed in which the number of points earned in the OSCE in the anatomy part of the examination was compared with the number of points earned in the palpation technique part (see Fig. 3).

Across all groups, there was an association between palpation skills and anatomical knowledge, with a correlation coefficient of 0.656 (Pearson correlation) and 0.593 (Spearman correlation). The better the students’ anatomical knowledge, the better they performed the palpation techniques and vice versa.

**Fig. 4:**
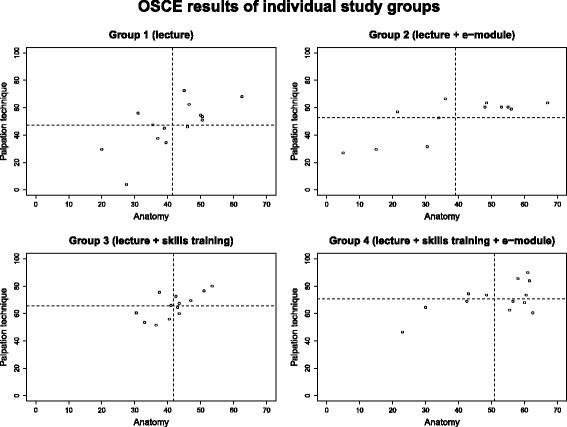
Correlation analysis between anatomical knowledge and palpation skills in the individual study groups based on the OSCE results

In order to determine the influence of the training seminar and the e-module on the observed association between anatomical knowledge and palpation skills, correlation analyses were performed in the individual study groups (see Fig. 4). Due to the small size of the study groups (*n* = 12–14) and the widely varying range of scores, it was not possible to compare the correlation coefficients in the individual study groups. Nevertheless, the point clouds could be described and interpreted. The point spread was highest within group 1 (lecture only) and group 2 (lecture and e-module). The spread in group 3 (lecture and skills training) was least pronounced. The mean point value for palpation skills was around 50 points for groups 1 and 2. The students’ palpation skills were improved the most through the skills training (by a mean value of approximately 70 points). Students in group 4, who had access to the e-module in addition to the skills training, showed significantly increased theoretical anatomical knowledge, by a mean value of 10 points.

### Evaluation of acceptance by students

The student evaluation yielded results that were similar to the results of the OSCE described above. Each student graded his or her randomly assigned study method with respect to how helpful it was for achieving the learning objectives in preparation for the examination, from 1 (*very good*) to 5 (*unsatisfactory*). The skills training − e-module combination received the highest grades (φ 1.28 ± 2.7).

The students developed a better appreciation of the clinical relevance of the anatomy course as a result of the skills training (90%) than with the lecture alone (20%). They rated their awareness of the clinical relevance of the learning content using a 5-level Likert scale from 1 (*definitely*) to 5 (*not at all*). The skills training received the highest rating for clinical relevance (φ 1.67 ± 0.866) and the clinical relevance of the training plus e-module received a rating of φ 2.18 ± 0.603.

## Discussion

### Anatomical learning in a clinical setting: the clinical relevance of palpation

Learning palpation techniques for the head and neck muscles as the first clinical skills acquired in the preclinical anatomy course allowed students without extensive preexisting knowledge of dentistry to learn a highly clinically relevant dental examination technique. Palpation of the head and neck muscles is one of the most basic diagnostic examinations for patients with suspected temporomandibular joint dysfunction (TMD). TMD is a general term for pain in the area of the temporomandibular joint (jaw) and the surrounding temporomandibular muscles. The pain is often caused by a myofascial dysfunction of extra-articular origin although stress is another possible cause [17]. In most cases, this leads to hyperactivity of the masticatory muscles. The most frequent symptoms of TMD are tension headaches and sensitivity to palpation in the area of the jaw [18–20].

Chinnah, De Bere, and Collett [11] surveyed students about the impact of learning anatomy in the clinical context of a peer physical examination course on their attitude toward the course material and their learning outcomes. The students emphasized that the clinical relevance of anatomy generally, and its significance for their future work in particular, had become much clearer. This in turn raised their intrinsic motivation, enhanced self-confidence, and ultimately increased learning outcomes [11]. Other authors have reported similar results [5, 6, 21].

In our study, the students indicated that the skills’ training was the method that most clearly illustrated the clinical relevance of palpation skills.

### Palpation skills

An understanding of TMD and the corresponding anatomical knowledge are prerequisites for a thorough and successful physical examination of the affected patient [20]. The clinician must have detailed knowledge of muscle location, insertion, and origin. This knowledge is also essential for improving clinical skills [17, 22].

The skill or technique of palpation was taught in the course clearly (peer teaching, videos) and easily. The clinical approach used for the examination corresponds to the approach called for by the German Society of Dentistry and Oral Medicine (DGZMK) [20, 22].

### Teaching method: blended-learning peer teaching

In medicine and in the teaching of anatomy in particular, the use of peer teachers has been common practice for many years [11, 23–25]. As a result of our intensive 2-h training course for the peers, there was no need for intervention on the part of the instructors during the student training sessions as has been reported and discussed in the literature before [26, 27]. The peers, who were well versed in the Peyton method, were able to access the e-module at all times during the seminar, which allowed them to be more self-assured as trainers and reinforced their own skills [28–33]. Our students accepted the peer assistants as full-fledged instructors, a phenomenon previously described in the literature [25]. In this respect, “medical resources” could definitely be saved during the training course.

### Enhanced knowledge and skills

The course was structured to allow students to achieve their learning objectives. For this reason, formulating the learning objectives in advance was an important step for developing the course concept. Correlation analyses clearly showed the reciprocal influence of theoretical knowledge and practical skills on learning outcomes. While palpation skills were improved by the training in particular, the e-module alone did not produce any significant enhancement of learning outcomes. However, when the e-module was combined with the skills training, the learning outcomes were greatly improved. Moreover, a significant increase in anatomical knowledge was observed. When anatomical knowledge was conveyed parallel to the palpation training, students were able to consolidate it through the simultaneous application of the skills, as was shown by the results. The students were highly motivated by the hands-on practice at the dental clinic and the relevance to everyday dental practice.

The results of our study are a good example of the efficiency of combining face-to-face teaching and e-learning (blended learning) and they reflect the findings of many other studies [6, 32, 34, 35].

### E-module

Today’s students are members of a new generation of “digital natives”, for whom using interactive media is second nature. Furthermore, they do not view the use of electronic media in teaching as a privilege but rather as their right [36]. In recent years, therefore, various forms of electronic media have become increasingly relevant in education because of the many ways they can be used [37], and this has ultimately changed teaching itself [12, 38, 39].

The increasing use of interactive media in teaching has changed the role of the instructor as well. In face-to-face courses, expert discussion of students’ questions on details is increasingly predominating and is replacing the imparting of basic knowledge [2, 35]. The instructor is now tasked with selecting good resources and relevant information from a vast pool of material. E-learning must be appreciated as a tool that can also support instructors in face-to-face teaching [38]. For successful implementation and, in turn, to positively influence the students’ learning outcomes, it is important for teachers to also use interactive media in face-to-face courses [36]. This means that the teaching staff is directly dependent on the efficiency of the tools [38].

After observing and evaluating web-based teaching for more than six years, McNulty, Sonntag and Sinacore concluded that the use of interactive media is at least equivalent and in some cases even superior to conventional teaching methods with respect to the satisfaction of the students and the learning effect [37]. Various authors have also demonstrated that combining web-based teaching with traditional teaching methods can improve learning outcomes [2, 35, 40]. In dental training, too, the use of interactive media or e-learning components is increasingly common. The observed acceptance on the part of the students is very high, and they achieve equivalent or better results in examinations than they do with teaching without web-based media [36].

The use of the e-module in the context of our project was also very well-received by the students. In the evaluation, they expressed their wish to use more electronic media in their studies. The e-module allowed them to further enhance the high learning outcomes of the skills training despite the fact that the e-module alone did not demonstrate any visible learning effects.

When we were developing the e-module, standardizing the learning content and developing the teaching material turned out to be the most time-consuming tasks. But these steps are crucial. If the teaching content is not standardized and thus not valid for various medical disciplines, especially in terms of practical skills, then the students will constantly be confronted by the fact that the same skill is taught differently in different disciplines within the same medical school. This will obviously lead to confusion and dissatisfaction. On the other hand, developing and standardizing the teaching material are tasks that need to be performed only once, before the course is first conducted. The materials can then be reused the following year and the students can use it in their clinical part of their studies. In our case, we were able to produce high-quality teaching material that could even be used as reference material in the regular dental clinical courses.

### Interdisciplinarity

Improving communication between disciplines is an important prerequisite for interdisciplinary projects and for standardizing procedures. It allows us to better coordinate content and permits us to set more plausible priorities with clinical relevance, which in turn helps us avoid redundancies. This joint project between the preclinical section and the clinical section improved the student’s motivation and learning outcomes. The students gave it high ratings. Ultimately, the project allowed the current requirements of the German Medical Licensure Act and the draft of the Dental Licensure Act to be met: Teaching in the interdisciplinary head-and-neck anatomy course promoted interdisciplinary thinking; it was purposeful during the palpation course component; and it had a problem-solving orientation. From the beginning of the preclinical second year on, theory and clinical knowledge were intensively linked [10].

### Limitations of the study

There are some limitations of our study which should be discussed. Group 4 which had a 2-h skills training additionally and free access to the e-module had more cumulative intervention time as the other groups. This might bias the results in the OSCE examination, in which both the anatomy and the palpation skills have been tested. On the other hand, the other groups had the same time to practice on their own and to get the anatomical knowledge; group 2 also in addition with the e-module.

A further limitation is the time point of OSCE assessment already two days after the intervention. To test whether the new course has really improved the students’ anatomical knowledge and palpation skills for their clinical years, a second OSCE examination has to be performed for further research.

## Conclusions

The “Palpation of the Head and Neck Muscles” interdisciplinary skills training course linking basic anatomical knowledge and clinical skills led to clearly improved learning outcomes for both anatomical knowledge and clinical skills. The additional use of an e-learning tool in combination with the skills training improved the learning effect, although the e-module alone did not produce any significant enhancement of learning outcomes.

Correlation analyses showed the reciprocal influence of theoretical knowledge and practical skills on learning outcomes. The better the students’ anatomical knowledge, the better they performed the palpation techniques and vice versa.

The students were highly motivated by the hands-on practice at the dental clinic and the relevance to everyday dental practice. Therefore, the students’ perception of the clinical relevance of the learning has been increased.

The interdisciplinary project demonstrated a successful connection between preclinical and clinically relevant teaching. A direct link was established between basic anatomical knowledge and clinical skills. A structured approach allowed the existing curriculum to be expanded by the important aspect of clinical relevance for instructors and students, and in so doing, significantly improved the students’ learning outcomes. Further research is required to evaluate the long term benefit of the new course for the clinical work.
